# Infectious disease specialist consultations in a Japanese cancer center: a retrospective review of 776 cases

**DOI:** 10.1186/s12913-020-05380-6

**Published:** 2020-06-03

**Authors:** Naoya Itoh, Yoshiro Hadano, Yasumasa Yamamoto, Norihiko Terada, Hanako Kurai

**Affiliations:** 1 Division of Infectious Diseases, Shizuoka Cancer Center Hospital, 1007 Shimonagakubo, Nagaizumi, Sunto-gun, Shizuoka, 411-8777 Japan; 2 Department of Infection Control and Prevention, Tokyo Medical and Dental University Hospital, Bunkyo-ku, Tokyo, Japan

**Keywords:** Cancer, Infectious diseases, Consultation services, Cancer care facilities, Japan, Retrospective review

## Abstract

**Background:**

Little is known about the impact of infectious disease (ID) consultations on the management of patients with cancer. This study aimed to describe the consultation services provided by ID specialists to all departments in a comprehensive cancer center in Japan.

**Methods:**

We conducted a retrospective review of ID consultations with adult patients at a comprehensive cancer center in Japan from April 2017 to March 2018.

**Results:**

During the study period, 776 patients with cancer had an ID consultation. Of these, 414 (53.4%) were hospital inpatients. Reasons for the ID consultation comprised clinical management (*n* = 481, 62.0%), immunization (*n* = 272, 35.1%), and infection control (*n* = 23, 3.0%). Of the 474 ID consultations for diagnostic purposes, the most frequent condition was fever or elevated inflammatory markers of unknown origin (*n* = 125, 26.4%). The most frequent diagnoses after the diagnostic ID consultation were hepatobiliary infections (*n* = 97, 22.4%), respiratory infections (*n* = 89, 20.618.8%), and intra-abdominal infections (*n* = 71, 16.4%). The commonest reasons for immunization consultations were to prevent seasonal influenza (*n* = 193, 71.0%) and post-splenectomy vaccination (*n* = 58, 21.3%). The commonest reasons for infection control consultations were suspected tuberculosis or contact with tuberculosis (*n* = 11, 47.8%) and herpes zoster infection (shingles) (*n* = 7, 30.4%).

**Conclusions:**

ID specialists play an important role in the clinical management of patients with cancer. ID physicians who work in cancer centers need to be specialized in treating IDs, diagnosing the causes of fevers of unknown origin, and controlling infection.

## Background

Infections are amongst the commonest complications of cancer, as cancer therapies, such as surgery, chemotherapy, and radiation therapy, cause immunodeficiencies. In patients with cancer, the appropriate diagnosis and management of an infection is often challenging because infections frequently have subtle or atypical manifestations [1]. Therefore, infectious disease (ID) specialists can contribute to the diagnosis and management of infections in patients with cancer [2, 3]. However, there are very few ID specialists in Japan (0.9 per 100,000 persons in Japan, compared to 2.4 per 100,000 persons in the USA) [4]. As of May 2019, there were only 1493 board-certified ID specialists in Japan (Japanese Association for Infectious Diseases data).

Previous studies have found that ID consultations play a positive role in the management of patients with other, non-cancerous conditions, and are generally associated with good patient outcomes [5–8]. Globally, however, there have been very few studies published on ID consultations among patients with cancer. A previously published retrospective review of inpatient ID consultations requested by surgeons at our cancer center found that numerous surgeons were in favor of ID specialists assuming a more direct role in the care of difficult cases [1]. However, this previous study was limited in scope and did not include outpatients, patients from all departments, or ID consultations for reasons other than a clinical diagnosis and management. Therefore, the aim of the present study was to describe the consultation services provided by ID specialists to all departments in a comprehensive cancer center in Japan.

## Methods

### Study design and patient population

We conducted a retrospective observational study at the Shizuoka Cancer Center Hospital, a 615-bed tertiary care hospital, in which approximately 8000 patients per year are admitted to surgical departments and approximately 7000 are admitted to internal medicine departments. The surgical departments comprise neurosurgery, head and neck surgery, thoracic surgery, esophageal surgery, gastric surgery, colorectal surgery, hepato-biliary-pancreatic surgery, breast surgery, gynecology, urology, ophthalmology, dermatology, plastic surgery, orthopedic surgery, and dental surgery. The internal medicine departments comprise gastroenterological medicine, respiratory medicine, hematology, endoscopy, palliative medicine, radiology, female internal medicine (breast oncology), cardiology, rehabilitation, neurology, and ID. At the time of this study, the ID team comprised four full-time ID specialists and four fellow doctors. The ID team provides inpatient and outpatient ID consultations to prevent, diagnose, and treat IDs in adult patients with cancer. It is institutional policy that the ID Department should be consulted for all vaccinations, including post-transplant, post-splenectomy, seasonal influenza, and other catch-up vaccines, and that all vaccinations be performed in the ID Department. We extracted data from the ID consultation database for all formal ID consultations from April 1, 2017 to March 31, 2018. Informal consultations and follow-up ID consultations were excluded.

### Data collection

The following patient data was extracted from the cancer center’s ID consultation database: age, sex, place of consultation (inpatient or outpatient), type of ID consultation (clinical consultation, immunization, or infection control), original reason for referral at the time of the first ID consultation, the referring division, type of cancer, stage of disease, antimicrobial use within 1 month after clinical consultation, death within 30 days after the ID consultation, change in antimicrobial practice after ID consultation, history of bone marrow transplant, changes in antimicrobial practice among cancer patients after the ID consultation, post-bone marrow transplant and post-splenectomy immunizations, and the type of vaccine, if applicable. The original reasons for the ID consultation were categorized as for diagnosis and management purposes, or for surgical antimicrobial prophylaxis.

### Data analysis

All analyses were performed using Excel Version 1904 (Microsoft Corporation, Redmond, WA, USA).

## Results

During the study period, the total numbers of inpatients and outpatients were 15,070 and 294,922, respectively. Overall, 776 patients were referred to the ID Department. The patients had a median age of 68 years (range: 18–92 years, interquartile range: 59–75 years). Most of the patients (64.4%) had advanced cancer (Stages III or IV). The other demographic and clinical characteristics of the study population are shown in Table 1. The majority of consultations (62.0%) were clinical in nature, and the majority of patients (89.8%) had solid tumors.

**Table 1:** Patient demographic and clinical characteristics (*N* = 776)

Characteristic	Patients ***n*** (%)
**Sex**	
Male	448 (57.7)
Female	328 (42.3)
**Place of consultation**	
Inpatient	414 (53.4)
Outpatient	362 (46.6)
**Type of consultation**	
Clinical consultation^a^	481 (62.0)
Immunization^b^	272 (35.1)
Infection control^c^	23 (3.0)
**Underlying cancer**	
Hematologic malignancies	42 (5.4)
Underwent a bone marrow transplant	21 (2.7)
Solid tumors	697 (89.8)
*Digestive tract*	422 (54.4)
*Respiratory tract and thoracic cavity*	68 (8.6)
*Female reproductive organs*	71 (9.1)
*Lip, oral cavity, and pharynx*	52 (6.7)
*Breast*	33 (4.3)
*Urinary tract*	16 (2.1)
*Melanoma and other neoplasms of the skin*	9 (1.2)
*Eye, brain, and other parts of the CNS*^d^	8 (1.0)
*Other solid tumor*^e^	18 (2.3)
None	37 (4.8)
**Stage**^f^	
0	7 (0.9)
I	69 (8.9)
II	109 (14.0)
III	135 (17.4)
IV	364 (47.0)
Unknown	2 (0.1)
Antimicrobial use within 1 month after clinical consultation	328 (42.3)

^a^See Table 2; ^b^See Table 3; ^c^See Table 4; ^d^CNS: central nervous system

^e^The 18 other types of solid tumor were cancers of the prostate (*n* = 7); mesothelial and soft tissue (*n* = 5); cancer that was classified as ill-defined, other secondary, or cancer of an unspecified site (*n* = 3); cancer of the thyroid and other endocrine glands (*n* = 2); and one cancer of the bone and articular cartilage

^f^Stage was documented in 686 patients: The rest of the patients were not stratified because they had hematologic malignancies, malignancies of the CNS, and unclassifiable neoplasia

Clinical consultations were the commonest type of ID consultation. The reasons for requesting a clinical consultation are shown in Table 2 and were for diagnosis and management purposes in 474 (98.5%) cases and for surgical antimicrobial prophylaxis in 7 (1.5%) cases. The commonest reason for requesting a clinical ID consultation was for the diagnosis and management of fever or elevated inflammatory markers of unknown origin.

**Table 2:** Reasons for clinical consultations (*N* = 481)

Reason for consultation	Patients ***n*** (%)
**Diagnosis and management**	474 (98.5)
Fever or elevated inflammatory markers of unknown origin	125 (26.0)
Positive blood culture	84 (17.5)
Established infection	77 (16.0)
Respiratory infection	63 (13.1)
Critical condition	18 (3.7)
Febrile neutropenia	10 (2.1)
Lymphadenopathy	8 (1.7)
Opportunistic infection or multidrug-resistant infection	4 (0.8)
Infection unresponsive to broad-spectrum antibiotics	4 (0.8)
Diarrhea	2 (0.4)
Other	76 (15.8)
**Surgical antimicrobial chemoprophylaxis**	7 (1.5)

Of the 776 ID consultations, 272 (35.1%) were for immunizations. The reasons for immunization are shown in Table 3.

**Table 3:** Reasons for immunization (*N* = 272)

Reason for immunization	Patients ***n*** (%)
Seasonal influenza	193 (71.0)
Splenectomy	58 (21.3)
Bone marrow transplant	13 (4.8)
Other^a^	8 (2.9)

^a^The other immunizations comprised pneumococcal vaccine (*n* = 7) and rubella vaccine (*n* = 1)

The reasons for infection control consultations are shown in Table 4.

**Table 4:** Reason for the infection control consultations (*N* = 23)

Reason for consultation	Patients ***n*** (%)
Herpes zoster infection (shingles)	7 (30.4)
Suspected tuberculosis	6 (26.1)
Exposure to tuberculosis	5 (21.7)
Other^a^	5 (21.7)

^a^The other reasons for requesting an infection control consultation comprised influenza (*n* = 2), exposure to influenza (*n* = 1), suspected methicillin-resistant *Staphylococcus aureus* colonization (*n* = 1), and conjunctivitis (*n* = 1)

Of the 776 consultations, 314 (40.5%) were for patients in internal medicine departments and 462 (59.5%) were for patients in surgical departments (Table 5).

**Table 5:** Source of referral for patients who had an infectious disease consultation (*N* = 776)

Department and clinical section	Patients ***n*** (%)
**Internal medicine**	314 (40.5)
Gastroenterology	158 (20.4)
Respiratory medicine	60 (7.7)
Hematology	36 (4.6)
Endoscopy	18 (2.3)
Palliative medicine	16 (2.1)
Radiology	16 (2.1)
Other^a^	10 (1.3)
**Surgery**	462 (59.5)
Hepato-biliary-pancreatic surgery	153 (20.9)
Gynecology	76 (9.7)
Colorectal surgery	47 (6.1)
Gastric surgery	46 (5.7)
Head and neck surgery	26 (3.4)
Urology	22 (2.8)
Breast surgery	22 (2.7)
Thoracic surgery	16 (2.1)
Neurosurgery	16 (1.9)
Orthopedic surgery	11 (1.3)
Other^b^	27 (3.4)

^a^The sources of the other 10 consultations with patients from internal medicine departments were female internal medicine (*n* = 6), cardiology (*n* = 3), and rehabilitation (*n* = 1)

^b^The sources of the other 27 consultations with patients from surgical department were esophageal surgery (*n* = 9), dermatology (*n* = 8), plastic surgery (*n* = 6), and dental surgery (*n* = 4)

Of the 776 consultations, 474 (61.1%) were for diagnostic and management purposes. The final diagnoses of the patients who had ID consultations for diagnostic or management purposes are shown in Table 6. Of 125 consultations for fever or elevated inflammatory markers of unknown origin, 114 were infection cases and 11 were non-infectious cases, and none were classified as being of unknown etiology in the final diagnosis.

**Table 6:** Final diagnosis after clinical ID consultations (*n* = 474)

Final diagnosis	Patients ***n*** (%)
**Infectious conditions**	433 (91.4)
Hepatobiliary tract infections	97 (20.5)
Respiratory infections	89 (18.8)
Intra-abdominal infections^a^	71 (15.0)
Genitourinary infections	48 (10.1)
Skin and soft tissue infections	38 (8.0)
CRBSI^b^	32 (6.8)
Bone and joint infections	14 (3.0)
Gastrointestinal infection	12 (2.5)
Febrile neutropenia	8 (1.7)
Deep neck infections	7 (1.5)
CNS^c^ infections	6 (1.3)
Cardiovascular infections	3 (0.6)
Other	8 (1.7)
**Non-infectious conditions**	40 (8.4)
Cancer-associated^d^	16 (3.4)
Other	24 (5.1)
**Unknown**	1 (0.2)

^a^ The 71 patients with intra-abdominal infections included peritonitis or peritoneal abscess (*n* = 57), intra-tumor infection (*n* = 8), appendicitis (*n* = 4), and lymphocyst infection (*n* = 2)

^b^CRBSI: catheter-related blood stream infection; ^c^CNS: central nervous system

^d^ The 16 patients with cancer-associated diseases included cancer or metastasis (*n* = 9), and tumor fever (*n* = 7)

The 30-day overall mortality following an ID consultation was 6.3% (Table 7). The antimicrobial practice changed after ID consultations in 57.8% of patients.

**Table 7:** Patient outcomes related to clinical ID consultations (*n* = 474)

Clinical outcomes	Patients ***n*** (%)
All-cause mortality within 30 days after ID consultations	30 (6.3)
Change in antimicrobial practice after ID consultations	
Yes^a^	274 (57.8)
No^b^	200 (42.2)

^a^Changes included new, additional, changed, or discontinued antimicrobial agents

^b^Lack of change included continuation of antimicrobial drugs and only testing and advice

## Discussion

We reviewed 776 ID consultations among patients with cancer who underwent care at our cancer center. Although various studies have reported that ID consultations are associated with better outcomes in patient management [5–8], the literature on the role of ID specialists in a cancer center is scarce. To the best of our knowledge, there has been only one previous study [1] published on ID consultations among Japanese patients with cancer. However, this previous study, which was conducted in our cancer center, was restricted to surgical inpatients. This study is the first to address ID specialist consultations among both inpatients and outpatients in a comprehensive cancer center in Japan, and is also the first to describe ID consultations for infection control and immunizations among patients with cancer.

There were more requests for ID consultations from surgical departments than from internal medicine departments. From the time that the ID unit at our hospital was established in 2003, ID consultation referrals from surgeons have increased, suggesting that surgeons need the specialized knowledge and skills of ID physicians in surgical oncology settings [1]. In another study of all types of patients, conducted at another Japanese tertiary care hospital, the surgical department was also the department that most frequently requested ID consultations [9]. This may be because in surgical oncology, patients experience a wide variety of nosocomial infections, including surgical site infections. Thus, ID specialists may play a role in caring for patients with solid tumors, especially after the patients have had surgery.

Clinical consultations for diagnostic and management purposes were the commonest type of ID consultation. Of these consultations, the commonest reason for requesting the consultation was for diagnosing the cause of fever or elevated inflammatory markers of unknown origin. Because infections account for approximately 70% of nosocomial fevers, surgeons frequently request that ID physicians determine whether the fever is caused by an infection [10]. However, nosocomial fever has various causes other than infection. Of the 474 clinical ID consultations, 40 cases (8.4%) were classified being non-infectious in the final diagnosis. Because diagnostic divisions, such as general internal medicine, are rare in Japanese cancer centers, ID specialists are also required to assist with challenging nondiagnostic cases or make diagnoses in patients with conditions that are difficult to diagnose, such as those with a fever of unknown origin [9]. Hence, ID specialists may play a role in caring for patients who have conditions that are difficult to diagnose [5, 11].

Immunization was the second commonest reason for requesting an ID consultation. The most frequent reason for requesting an immunization consultation was to vaccinate patients against seasonal influenza. In our hospital, we advocate for all patients with cancer to be vaccinated against influenza annually, as patients with cancer who develop influenza are at increased risk of developing severe influenza-related complications, with a four-fold higher hospitalization rate, and a ten-fold higher mortality rate than that in the general population [12–15]. Furthermore, patients with cancer have an increased risk of developing influenza and influenza-related complications even if they have completed chemotherapy or are between courses of chemotherapy [13]. A study by Poeppl et al. [12], conducted in Austria, found that although annual vaccination against influenza was recommended, vaccination rates among patients with cancer were low. In the study by Poeppl et al., the main reasons for refusing influenza vaccination were concerns about the possibility of interactions between the vaccine and their cancer, and the possibility of side-effects. However, the study also showed that patients with cancer were more likely to accept influenza vaccination if it was recommended by their doctor. Therefore, ID specialists should recommend to physicians working in all divisions of the cancer center, that they vaccinate patients with cancer against influenza annually.

Although patients with hematologic malignancies are more vulnerable to infectious disease than are those with solid organ malignancies, most ID consultations were for patients with solid organ malignancies. Pongas et al. [3] reported that in their study, almost half of the patients had hematologic malignancies. Although the reason why patients with hematologic malignancies were in the minority in this study remains unclear, it may have been due to poor communication between our department and the hematology department.

This study has several limitations. First, it was a retrospective observational study that was conducted at a single center; thus, the study findings may not be generalizable to other settings. Second, because the study comprised a retrospective medical record review, we cannot exclude the possibility of classification biases and diagnostic errors associated with the ID consultations. Finally, we did not review the patients’ microbiology results.

## Conclusions

This study described the roles of ID specialists in a Japanese cancer center. It identified several key competencies that are needed by ID physicians who work in cancer centers. These include being specialized in treating IDs, diagnosing the causes of fevers of unknown origin, and controlling infection. We conclude that ID specialists might contribute to providing optimal patient care for individuals with malignancy.

## Data Availability

The datasets used during the current study are available from the corresponding author on a reasonable request.

## Abbreviations

ID: Infectious disease
